# Efficient manipulations of circularly polarized terahertz waves with transmissive metasurfaces

**DOI:** 10.1038/s41377-019-0127-0

**Published:** 2019-01-30

**Authors:** Min Jia, Zhuo Wang, Heting Li, Xinke Wang, Weijie Luo, Shulin Sun, Yan Zhang, Qiong He, Lei Zhou

**Affiliations:** 1 State Key Laboratory of Surface Physics and Key Laboratory of Micro and Nano Photonic Structures (Ministry of Education), and Department of Physics, Fudan University, 200438 Shanghai, China; 2 Beijing Key Laboratory of Metamaterials and Devices, Key Laboratory of Terahertz Optoelectronics (Ministry of Education), and Beijing Advanced Innovation Center for Imaging Technology, Capital Normal University, 100048 Beijing, China; 3 Shanghai Engineering Research Center of Ultra-Precision Optical Manufacturing, Green Photonics and Department of Optical Science and Engineering, Fudan University, 200433 Shanghai, China; 4 Collaborative Innovation Center of Advanced Microstructures, 210093 Nanjing, China

**Keywords:** Metamaterials, Terahertz optics

## Abstract

The unrestricted control of circularly polarized (CP) terahertz (THz) waves is important in science and applications, but conventional THz devices suffer from issues of bulky size and low efficiency. Although Pancharatnam–Berry (PB) metasurfaces have shown strong capabilities to control CP waves, *transmission*-mode PB devices realized in the THz regime are less efficient, limiting their applications in practice. Here, based on Jones matrix analysis, we design a tri-layer structure (thickness of ~λ/5) and experimentally demonstrate that the structure can serve as a highly efficient *transmissive* meta-atom (relative efficiency of ~90%) to build PB metadevices for manipulating CP THz waves. Two ultrathin THz metadevices are fabricated and experimentally characterized with a *z*-scan THz imaging system. The first device can realize a photonic spin Hall effect with an experimentally demonstrated relative efficiency of ~90%, whereas the second device can generate a high-quality background-free CP Bessel beam with measured longitudinal and transverse field patterns that exhibit the nondiffracting characteristics of a Bessel beam. All the experimental results are in excellent agreement with full-wave simulations. Our results pave the way to freely manipulate CP THz beams, laying a solid basis for future applications such as biomolecular control and THz signal transportation.

## Metasurfaces: efficient terahertz control

Ultrathin metasurfaces that can efficiently manipulate circularly polarized terahertz waves in transmission rather reflection have been demonstrated by scientists in China. The devices were fabricated by Min Jia and colleagues from Fudan University and Capital Normal University. The tri-layer structures operate at frequencies of around 0.6 THz and suit future on-chip applications for terahertz photonics. Various designs were tested resulting in a circularly polarized terahertz Bessel beam generator and a device exhibiting the photonic spin Hall effect. The metasurfaces consist of a periodic array of meta-atoms, each composed of three thin layers of metal in a U-shape that are separated and surrounded by polyimide. Changing the angular orientation of the meta-atoms allows different phase gradients to be programmed into the metasurfaces bringing the desired functionality.

## Introduction

The manipulation of circularly polarized (CP) terahertz (THz) waves in a predesigned manner is highly desired due to both curiosities in fundamental physics and pressing technological demands in applications. For example, as many biomolecules exhibit chiral structures with rotational/vibrational modes in the THz regime, they interact distinctly with CP THz beams depending on their handedness. Thus, using specific CP beams (such as Bessel beams (BBs)) to control the motions of such biomolecules is very promising in many applications, such as drug delivery and biological sensing^1,2^. In addition, handedness multiplexing can be a useful approach to increase the information processing capability of THz telecommunications. However, conventional THz devices (i.e., waveplates^3^, lenses^4^, and axicons^5^) typically suffer from issues of bulky size and/or low efficiency due to the weak interactions between THz waves and naturally existing materials^6^, which only exhibit electric responses.

Metasurfaces, ultrathin metamaterials that consist of planar subwavelength units (e.g., meta-atoms) with tailored electromagnetic (EM) responses, have demonstrated unprecedented capabilities in controlling EM waves^7–10^. By carefully designing metasurfaces with different phase and amplitude profiles for transmitted or reflected waves, scientists have realized many fascinating EM wave manipulation effects, such as anomalous refraction/reflection^11–14^, surface wave excitations^15–17^, metaholograms^18,19^, flat lenses^20–22^ and many others^23–25^. In particular, Pancharatnam–Berry (PB) metasurfaces^26,27^, constructed by *identical* meta-atoms with orientation angles rotated successively, exhibited exceptional abilities in manipulating CP light. Different from metasurfaces that control linearly polarized (LP) waves where local phases are typically dictated by structural resonances, PB meta-atoms acquire extra phases for CP waves from a geometrical origin^28–30^. Many PB metadevices have been proposed to control CP beams, yielding intriguing phenomena such as the photonic spin Hall effect (PSHE)^30–32^ and the generation of special beams (such as vortex^33^ or BBs^34^). Unfortunately, in the THz domain where functional devices are particularly lacking, we found that the realized PB metadevices are either inconvenient for practical applications in a reflection geometry^35–38^ or inefficient in transmission mode^39–41^. It was recently recognized that the working efficiency of a PB metadevice is inherently tied to the transmission/reflection Jones matrix of its constitutional meta-atom^30^. Although high-efficiency *reflective* PB meta-atoms are relatively easy to design and fabricate at frequencies ranging from the microwave to the visible regions, high-efficiency *transmissive* PB meta-atoms with *deep-subwavelength thicknesses* are very difficult to realize at frequencies higher than GHz, eventually due to the strict Jones matrix conditions required for transmission mode^31^.

**Fig. 1:**
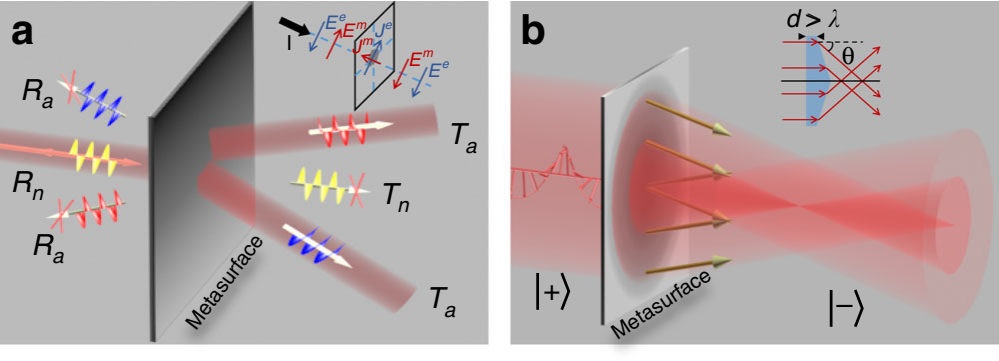
Working principle of the high-efficiency photonic spin Hall effect (PSHE) and background-free Bessel beam (BB) generation for circularly polarized (CP) waves in a transmission geometry. **a** Schematic of high-efficiency PSHE achieved by a transmissive Pancharatnam–Berry (PB) metasurface constructed by an appropriately designed meta-atom exhibiting both electric and magnetic responses as depicted in the inset. *R*_a_, *R*_n_, *T*_a_, and *T*_n_ represent the power efficiencies of the anomalous and normal modes on the reflection and transmission sides, respectively. **b** Schematic of background-free CP BB generation based on a high-efficiency PB metasurface. Here, $\left| + \right\rangle$ and $\left| - \right\rangle$ represent left and right circular polarizations, respectively. Inset: schematic of the working principle of a conventional axicon

In this article, we experimentally demonstrate that *high-performance transmissive* PB metadevices can be realized in the THz regime as long as an appropriate PB meta-atom is designed. Our meta-atom is a freestanding tri-layer structure with effective magnetic currents induced via interlayer couplings, which are crucial for satisfying the Jones matrix criteria^30,31^. After experimentally characterizing the Jones matrix properties of our PB meta-atom, we then employ the meta-atom as a building block to construct two ultrathin PB metadevices that can manipulate THz waves with high performance. Specifically, our experiments reveal that the first device can realize a PSHE (see Fig. 1a) with *undesired* modes significantly *suppressed*, yielding a measured relative efficiency of 90%, whereas the second device can generate a high-quality CP THz BB exhibiting desired nondiffracting properties *without* normal-mode background noise (see Fig. 1b). THz metadevices with such high performances have rarely been observed in the literature. Our fabricated devices are ultrathin (thickness ~λ/5) and flat and highly favorable for future on-chip applications, which are in sharp contrast to conventional devices (such as an axicon, inset of Fig. 1b) or dielectric metasurfaces with wavelength-scale thicknesses^33,42–45^. Our findings establish an ultrathin and flat platform to efficiently manipulate CP THz waves, which can stimulate further studies related to biomolecule control and sensing as well as THz signal transport.

## Results

### Design and characterization of the high-efficiency meta-atom

Suppose a planar meta-atom placed in an *xy*-plane exhibits an appropriate mirror symmetry such that its transmission/reflection characteristics can be described by two diagonal Jones matrices $$R = \left( {\begin{array}{*{20}{c}} {r_{xx}} & 0 \\ 0 & {r_{yy}} \end{array}} \right)$$ and $$T = \left( {\begin{array}{*{20}{c}} {t_{xx}} & 0 \\ 0 & {t_{yy}} \end{array}} \right)$$, with *r*_xx_, *r*_yy_, *t*_xx_, and *t*_yy_ denoting the reflection/transmission coefficients for the waves polarized along the *x* and *y* axes, respectively. Using such meta-atoms to design PB metasurfaces with certain functionalities (e.g., PSHE, focusing), our recent analyses^30,31^ indicated that such fabricated device can in principle create four different modes (see Fig. 1a, for the case of a PSHE metasurface), in which only one mode is responsible for the desired wave manipulation functionality (the anomalous transmission mode), whereas the other modes are either background noise (the normal transmission mode) or only take away energies (the anomalous/normal reflection modes). The power efficiencies of these four beams, denoted by *T*_a_, *T*_n_, *R*_a_, and *R*_n_, respectively, are determined by the Jones matrix elements of the meta-atom via

1 $$\begin{array}{l}T_a = \left| {\left( {t_{xx} - t_{yy}} \right)} \right|^2/4,\,R_a = \left| {\left( {r_{xx} - r_{yy}} \right)} \right|^2/4\\ T_n = \left| {\left( {t_{xx} + t_{yy}} \right)} \right|^2/4,\,R_n = \left| {\left( {r_{xx} + r_{yy}} \right)} \right|^2/4\end{array}$$

We first consider the ideal case neglecting losses. Obviously, to achieve a PSHE effect with 100% efficiency (i.e., *T*_a_ = 1), all the undesired modes should be completely suppressed (i.e., *R*_a_ = *R*_n_ = *T*_n_ = 0), yielding the following conditions

2 $$\begin{array}{l}\left| {r_{xx}} \right| = \left| {r_{yy}} \right| = 0,\,\left| {t_{xx}} \right| = \left| {t_{yy}} \right| = 1\\ \arg (t_{xx}) - \arg (t_{yy}) = \pi \end{array}$$

for designing our meta-atoms. Eq. (2) implies that the designed meta-atom should function as an ideal half-wave plate (HWP) with 100% transmittance. To design such a meta-atom, our previous analyses^31^ revealed that a single-layer resonator exhibiting *only electric* responses can never fulfill Eq. (2). We must search for meta-atoms simultaneously exhibiting *electric and magnetic* responses (see inset to Fig. 1a) with appropriate strengths for two different polarizations.

**Fig. 2:**
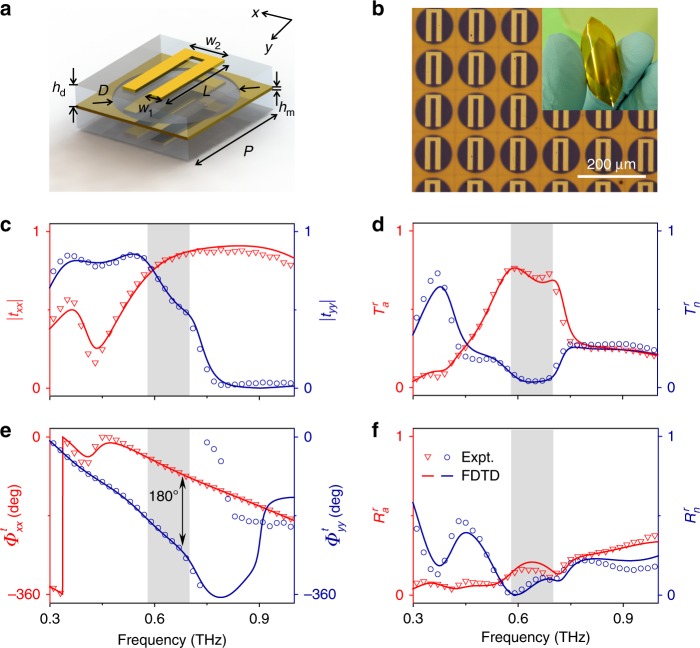
High-efficiency Pancharatnam–Berry (PB) meta-atom design and its optical properties. **a** Schematics of the designed high-efficiency PB meta-atom. **b** Optical image of part of the fabricated sample consisting of a periodic array of the designed meta-atoms. **c**, **e** Measured and simulated spectra of the transmission amplitude and phase for the fabricated sample illuminated by *x-* and *y*-polarized terahertz (THz) waves. Spectra of **d** $T_a^r,T_n^r$, and **f** $R_a^r,R_n^r$ of the PB meta-atom obtained from the experimental or simulated results of the Jones matrix characteristics. Here, the geometrical parameters of the meta-atom are *p* = 128 μm, *w*_1_ = 15 μm, *w*_2_ = 45 μm, *L* = 95 μm, *D* = 116 μm, *h*_*d*_ = 30 μm, and *h*_*m*_ = 65 nm

These considerations motivated us to design our meta-atom based on a freestanding anisotropic ABA structure^16,31,46,47^, which was proven to support the perfect transmission of EM waves under certain conditions. As shown in Fig. 2a, layer A in our meta-atom is a “U”-shaped metallic resonator, layer B is a metallic plate with holes loaded with the same “U”-shaped planar structure, and two 30-µm-thick polyimide spacers (*ε*_*r*_ = 3.1 + 0.04*i) are adopted to separate the two adjacent metallic layers. The interlayer couplings can create appropriate effective magnetic currents inside the structure, whereas the “U” shape provides enough freedom to generate lateral anisotropy in the EM responses. With careful structural tuning, we obtained the final design for our meta-atom and then fabricated a sample containing a periodic array (periodicity of 128 μm) of the designed meta-atoms by standard photolithography. Figure 2b depicts part of a top-view optical image of our fabricated sample, which is a freestanding membrane, as shown in the inset to Fig. 2b. In contrast to previous microwave design studies where metals have been considered as perfect electric conductors, here in designing our THz meta-atoms, material losses should be seriously considered in the optimization process. The additional geometrical freedom provided by the “U”-shaped resonator offers enough room to fine tune the responses of the whole device, yielding an optimized performance in terms of working bandwidth and efficiency (see Figs S1–S6 in Supplementary Information).

We then use a THz time-domain spectroscopy (TDS) system to characterize the Jones matrix properties of our fabricated sample. Figures 2c, e show the measured spectra of the transmission amplitude and phase of the sample for two orthogonal incident polarizations, respectively. Obviously, the designed meta-atom exhibits high transmission amplitudes but with a *π* phase difference for the two incident polarizations at a frequency interval centered at 0.6 THz (the shaded region in Fig. 2c, e). Note here that the peak transmission amplitudes cannot reach 100% due to material absorption. Simultaneously, the reflections from the meta-atom are significantly suppressed in the same frequency interval (see Fig. S7 in Supplementary Information), which together with Fig. 2c, e already imply that our designed meta-atom has high efficiency in the working frequency band. The high performance of our designed meta-atom can be more clearly seen in Fig. 2d, f, where the efficiency spectra of four modes are depicted. To better illustrate how the scattered energy distributes inside different modes, we purposely show in Fig. 2d, f the *relative* efficiencies of the four different modes, which are the ratios between the power flows carried by different beams and the sum of all scattered power (i.e., $T_j^r$ = *T*_j_/(*T*_a_ + *T*_n_ + *R*_a _+ *R*_n_), $R_j^r$ = *R*_j_/(*T*_a _+ *T*_n _+ *R*_a _+ *R*_n_), *j* = *n*, *a*), without taking absorption into account. At general frequencies, four beams can carry substantial portions of the scattered energy. However, in the working band, only the desired anomalous mode is alive while all other modes are significantly suppressed, implying the high performance of our meta-atom.

We also perform finite-difference time-domain (FDTD) simulations on realistic structures to understand the experimental results. As shown in Fig. 2c–f, all FDTD simulations are in good agreement with the measured experimental data. In addition to verifying the measurements, the FDTD simulations also reveal the physical mechanism responsible for the high performance of the designed meta-atom. Indeed, substantial magnetic currents are induced in the ABA structure (see Sec. 1 in Supplementary Information), which are crucial to yield high transmission of EM waves (and thus a high polarization conversion efficiency). In addition, the FDTD simulations reveal that material losses (especially metallic losses) are responsible for the nonideal performance of our PB meta-atom (see Figs. S8–S10 in Supplementary Information). With such a high-performance PB meta-atom, we can use it as a building block to realize many functional PB devices, with two examples presented in the following two subsections.

### High-efficiency PSHE

Utilizing our designed meta-atom as a building block, we first design a series of PB metasurfaces supporting a high-efficiency PSHE. As argued in^30,31^, for a CP wave with spin *σ* (*σ* = 1 denotes left circular polarization, whereas *σ* = −1 denotes right circular polarization) that is incident on a meta-atom with principle axes rotated by an angle *ϕ* relative to the *z* axis, the spin-reversed components of the waves scattered by the meta-atom will acquire an extra phase factor $e^{i\Phi_{\sigma}}$ with Φ_*σ*_ = *σ*·2*ϕ*. Therefore, to design a PSHE metasurface, one simply arranges the orientation angle *ϕ*(*x*) of the meta-atom located at position *x* to linearly depend on *x* (i.e., *ϕ*(*x*) = *ϕ*_0 _+ *ξ*·*x*/2) so that the phase profiles of the *anomalous* transmission components exhibit *opposite* phase gradients depending on the input spin: Φ_*σ*_(*x*) = Φ_0_ + *σ*ξ·*x*. Therefore, by illuminating the metasurface with an LP wave at an incident angle *θ*_*i*_, two anomalous beams will be generated on the transmission side traveling in two different directions dictated by

3 $$\theta _r^\sigma = \sin ^{ - 1}(\sin \theta _i - \sigma \xi /k_0)$$

with *k*_0_ = *ω*/*c* being the free-space wavevector. Note that the anomalous beams carry *opposite* spins with respect to their corresponding incident beams. Meanwhile, in general, there should also exist a normal-mode beam traveling in the same direction as that of the incident beam, with a power efficiency given by *T*_n_.

**Fig. 3:**
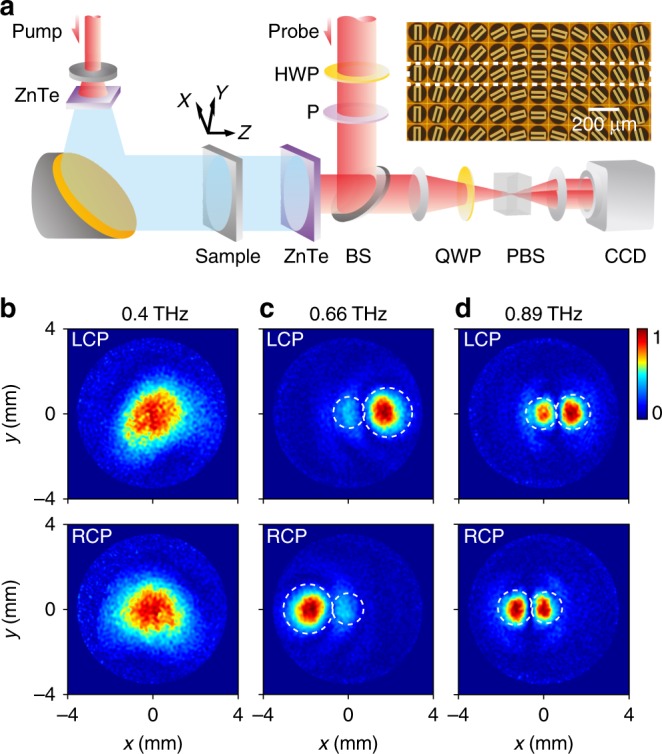
Experimental setup and characterization of high-efficiency photonic spin Hall effect (PSHE) in the terahertz (THz) regime. **a** Schematics of the THz digital holographic imaging system (inset: optical image of part of a fabricated Pancharatnam–Berry (PB) metasurface with *ξ* = 0.296*k*_0_). **b-d** Measured E field distributions of the transmitted LCP (top panel) and RCP (bottom panel) field components in an *xy*-plane located 3.5 mm away from the PB metasurface with *ξ* = 0.296*k*_0_, illuminated by normally incident LP beams at frequencies of 0.4 THz, 0.66 THz and 0.89 THz, respectively. The circles define the areas where integrations are performed to obtain the powers carried by different modes

We fabricate three THz PB metasurfaces with different phase gradients (*ξ* = 0.395*k*_0_, 0.296*k*_0_, 0.222*k*_0_) based on our designed meta-atom (see Fig. 3a and Fig. S13 (a-b) for their optical images) and then experimentally characterize their PSHE properties with our THz digital holographic imaging system (TDHIS) (see Fig. 3a). In our experiments, by illuminating the metasurfaces with *x*-polarized THz waves at different frequencies, we first obtain all the local **E** field information (with amplitude and phase) in LP bases in an *xy*-plane 3.5 mm away from the device and then transform the measured data to CP bases via *E*^σ^ = (*E*_x_ − *iσE*_y_)/$\sqrt 2$ to obtain the field components carrying different spins. Figure 3b–d show the measured spin-dependent **E** field distributions in the target *xy*-plane for one fabricated PB metasurface with *ξ* = 0.296*k*_0_ at three representative frequencies of 0.4 THz, 0.66 THz, and 0.89 THz, respectively. All the data are normalized with respect to a reference, which is the maximum value within a given pattern (see Fig. S11 in Supplementary Information for the original experimental data in an LP basis at 0.66 THz). At 0.66 THz within the working band, Fig. 3c clearly shows that the transmitted left circularly polarized (LCP) and right circularly polarized (RCP) waves have been predominantly deflected away from the central direction in two opposite directions, whereas only very weak signals appear at the central position corresponding to the normal transmission modes. The high contrast between the field strengths in the two circles already implies the high working efficiency of our device. Outside the working band, however, our PB metasurface always generates strong normal modes. At a low frequency (0.4 THz), the generated normal mode constitutes nearly all of the power of the transmitted wave, as shown in Fig. 3b. Note that the beam size of the input THz wave increases as the frequency decreases (see Fig. S12 in Supplementary Information).

**Fig. 4:**
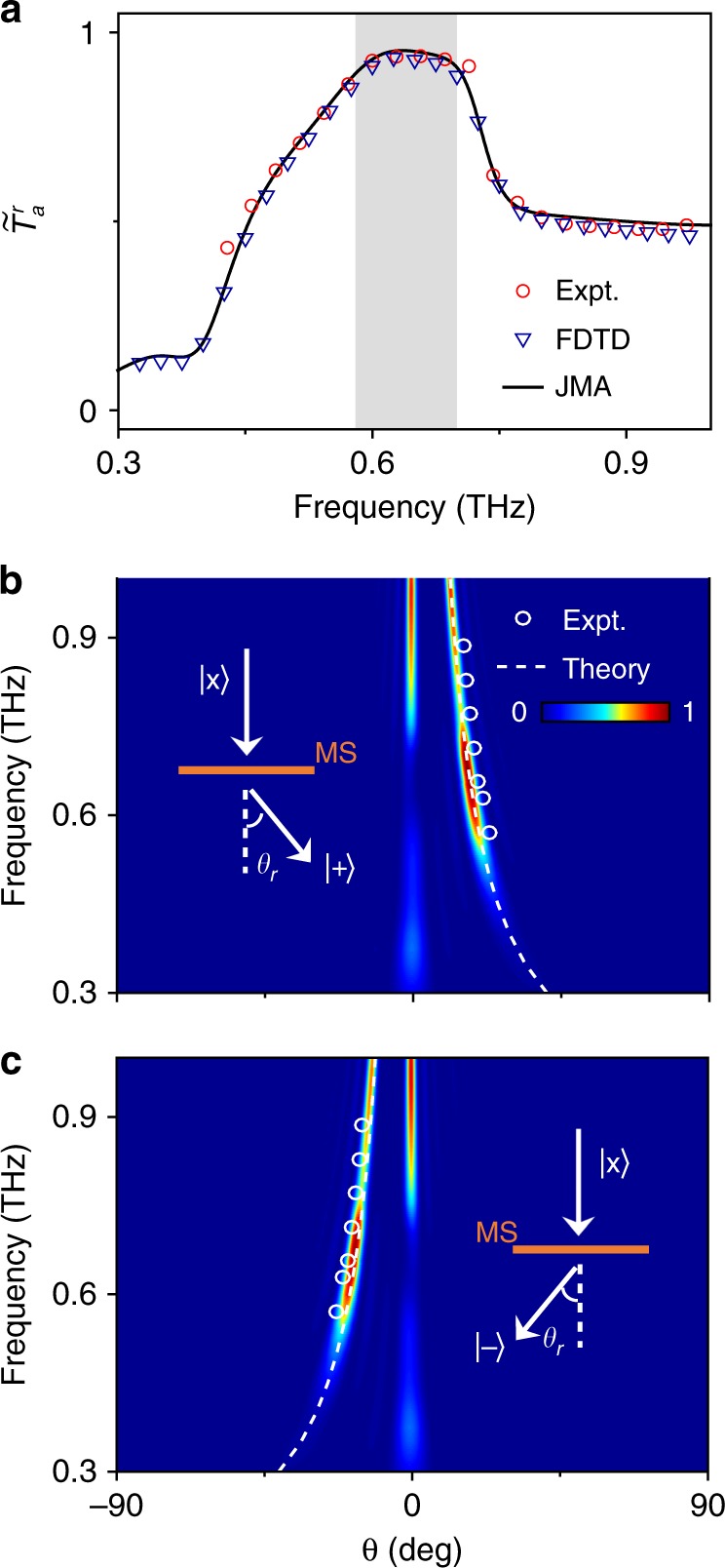
Performance of the realized photonic spin Hall effect (PSHE). **a** Spectra of the relative working efficiency $\tilde T_a^r$ of our device on the transmission side obtained by experiments, finite-difference time-domain (FDTD) simulations and Jones matrix analysis (JMA). The shaded region corresponds to the working band with $\tilde T_a^r$exceeding 80%. FDTD simulated scattered field intensity (color map) of the transmitted **b** LCP and **c** RCP waves versus frequency and deflection angle for the *ξ* = 0.296*k*_0_ PB metasurface illuminated by normally incident LP terahertz (THz) beams. The white circles and dashed lines in (**b**) and (**c**) represent the experimental results (from Fig. 3) and theoretically calculated results based on the generalized Snell’s law (Eq. (3))

To quantitatively evaluate the working efficiency of our fabricated device, we integrate the measured |*E*^*σ*^|^2^ inside the two circles corresponding to two anomalous modes with different spins and define the obtained value as (unnormalized) *T*_a_ and repeat the integrations over the two central circles to obtain (unnormalized) *T*_n_. Unfortunately, unlike the TDS system used to characterize the meta-atom properties (Fig. 2), here, our TDHIS system does not allow us to measure the reflected THz signals; thus, we cannot obtain the experimental data on *R*_a_ and *R*_n_. Therefore, we define a new physical quantity as the ratio between the power flows carried by the abnormal transmission mode and the total transmission power (i.e., $\tilde T_a^r$ = *T*_a_/(*T*_a_ + *T*_n_)), which can quantitatively evaluate the performance of our PB metasurface at the transmission side. The open circles in Fig. 4a depict the experimentally obtained $\tilde T_a^r$ as a function of frequency, showing that our PB metasurface can exhibit a maximum relative efficiency of 90% at 0.66 THz. We also performed FDTD simulations on realistic structures, from which we quantitatively evaluated the relative efficiencies at different frequencies. The $\tilde T_a^r$ spectra obtained by the FDTD simulations and the Jones matrix analysis (JMA) are compared with the experimental data in Fig. 4a. Excellent agreements are noted for these results.

The spin-dependent anomalous refractions enabled by our PB metasurface satisfy the generalized Snell’s law (Eq. (3)). The color maps in Fig. 4b, c illustrate the FDTD simulated scattering power versus frequency and refraction angle at the transmission side, respectively, measured by CP detectors with different spins, for our *ξ* = 0.296*k*_0_ metasurface illuminated by normally incident LP waves. Clearly, at general frequencies, both normal and anomalous modes appear at the transmission side (consistent with Fig. 1a). However, in the working band (0.58–0.7 THz), the FDTD simulations show that the strength of normal transmission is significantly suppressed with a strongly enhanced anomalous mode strength, again reinforcing our notion of high efficiency, as shown in Fig. 4a. The experimentally measured angles of spin-dependent anomalous refraction at different frequencies are denoted by open circles in the same figure, which match very well with the angles where the maximum transmission signals are detected in the FDTD simulations. The $\theta _r^\sigma$∼*f* relations, obtained by both experiments and FDTD simulations, are in perfect agreement with the theoretical predictions (dotted lines) given by Eq. (3) (setting *θ*_*i*_ = 0°). Similar conclusions hold for the other two samples fabricated (see Figs. S13 and S14 in Supplementary Information). Finally, we note that the anomalous refraction angle $\theta _r^\sigma$increases as the frequency *f* decreases, consistent with Eq. (3).

### Background-free CP BB generation

Recently, BBs have attracted intensive research interest due to their unique nondiffracting and self-healing properties. In particular, CP BBs in the THz regime are particularly useful for controlling the motion of chiral biomolecules by exerting optical forces, which can be attractive or repulsive depending on the details of the beam and the objects as predicted by recent theories^48,49^. However, the experimental generation of high-quality THz BBs with an ultrathin device has rarely been observed, especially in a *transmission* geometry favored for realistic applications. In this section, we utilize our meta-atom to construct another PB metadevice that can generate high-quality CP THz BBs with high efficiencies and without normal-mode background interference.

**Fig. 5:**
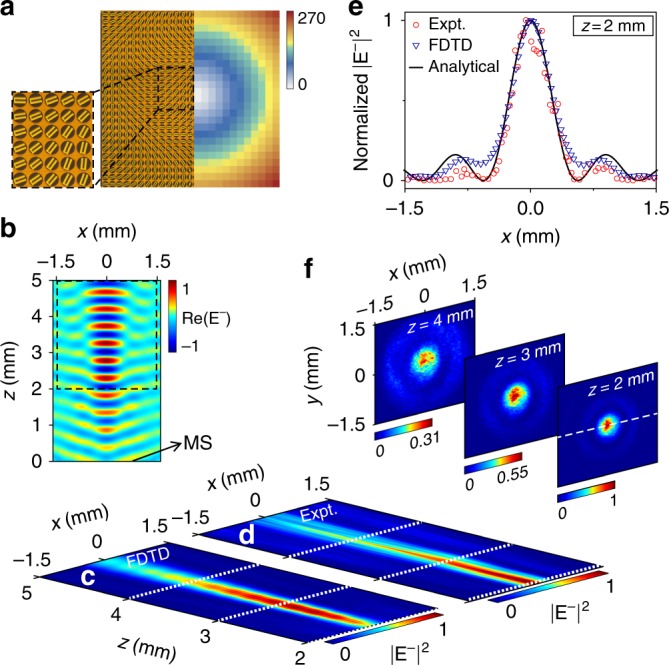
Design and characterization of background-free Bessel beam (BB) generation for circularly polarized(CP) terahertz (THz) waves. **a** Optical image of part of a fabricated CP BB generator, which is a Pancharatnam–Berry (PB) metasurface (left panel) designed based on a particular transmission-phase distribution (right panel). **b** FDTD simulated Re(*E*^−^) distribution in the xz plane with *y* = 0 mm for our metasurface (placed at *z* = 0 mm) illuminated by a normally incident *x*-polarized THz beam at 0.66 THz. **c** FDTD simulated and **d** z-scan measured |*E*^−^|^2^ distributions inside the area surrounded by black dashed lines in (**b**), under exactly the same conditions as in (**b**). **f** Measured |*E*^−^|^2^ distributions in xy planes with *z* = 2, 3, and 4 mm. **e** Normalized |*E*^−^|^2^∼*x* distributions along the line with *z* = 2 mm and *y* = 0 mm, obtained by the experiment (red circles), FDTD simulations (blue triangles) and theoretical formula for a zero-order BB (solid line)

A zero-order BB can be described by $E_{BB}(x,y,t) = e^{ik_zz} \times {\int}_0^{2\pi } {e^{ik_{||}(x\cos \varphi + y\sin \varphi )}\frac{{d\varphi }}{{2\pi }}} \times e^{ - i\omega t}$, where $k_{||}^2 + k_z^2 = k_0^2$ and *φ* denote the orientation angle of $\vec k_{||}$. In a conventional approach, an axicon is used to bend incident waves at an angle *θ* towards the optical axis of the device. The beam generated from the interference of locally transmitted waves well represents a BB, as shown in the inset to Fig. 1b. However, such a device is too bulky and inefficient for integrated optics applications. Here, we design a PB metadevice exhibiting a transmission-phase profile of Φ(*x*, *y*) = *k*_||_$\sqrt {x^2 + y^2}$ (see right panel in Fig. 5a) for input LCP waves, for which the orientation angles of the involved PB meta-atoms are set as *ϕ*(*x*, *y*) = Φ(*x*, *y*)/2. Such a PB device, which is flat and ultrathin, can well mimic an axicon to bend an incident LCP wave to an appropriate angle on the transmission side, thus generating the desired CP BB.

We first employ FDTD simulations to illustrate the performance of our designed PB device. Consistent with our experimental characterizations discussed later, we assume that our metadevice is illuminated by an *x*-polarized normally incident THz beam at 0.66 THz and then employ FDTD simulations to compute the distribution of Re(*E*^-^) (i.e., the RCP field component) in the *xz* plane with *y* = 0 mm on the transmission side. Figure 5b clearly shows that the RCP beam generated in this configuration is indeed a well-behaved BB exhibiting a clear nondiffracting feature. This finding is surprising at first glance. As the incident *x*-polarized THz beam contains both LCP and RCP components, after passing through our metadevice, in principle on the transmission side, there should appear both the desired RCP BB (the anomalous mode) and a normal-mode background. However, the pattern with a clean BB signature, as shown in Fig. 5b, already implies that the normal-mode background is very weak in this case, reinforcing our notion of a high working efficiency. The high extinction ratio between the desired BB and the undesired background is more clearly seen in Fig. 5b and Fig. S16(a), where the field patterns of the RCP and LCP components are directly compared on the transmission side of the metadevice, which is illuminated by an LP normally incident THz beam at 0.66 THz.

We next fabricate the PB metadevice shown in part in the optical image in the left panel of Fig. 5a and then experimentally characterize the performance of the BB generation with our TDHIS. Illuminating our metadevice with a normally incident *x-*polarized THz beam, we measure the amplitudes and phases of the Ex and Ey components of the transmitted THz wave at different *z* positions and then reconstruct both the *E*^+^ and *E*^−^ field components from the measured data. Due to the limitation of our *z*-scan system, we can only measure the field distributions inside the area surrounded by dashed lines in Fig. 5b. Figure 5d shows the measured intensity profile for the transmitted RCP beam (|*E*^−^|^2^) at 0.66 THz in the *xz* plane (*y* = 0 mm), which is in good agreement with the corresponding FDTD results (Fig. 5c). As the z-scan step in our measurement is 0.5 mm, which is not fine enough to clearly resolve the phase information of the generated BB (see Fig. 5b), we chose to depict the measured intensity pattern in Fig. 5d. Both Fig. 5c, d clearly illustrate the nondiffracting features of the generated BB. To characterize the performance of the generated RCP BB, we also experimentally measured the intensity distribution of the RCP component (|*E*^−^|^2^) in three *xy* planes at different longitudinal positions (*z* = 2, 3, and 4 mm). As shown in Fig. 5f, the generated transverse field patterns exhibit nice rotationally invariant symmetries with strengths that decay quickly away from the center. In Fig. 5e, we compare the intensity profiles along the *x* axis (with *z* = 2 mm and *y* = 0 mm), obtained by the experimental measurements, the FDTD simulations, and the theoretical formula for the desired zero-order BB. Excellent agreement among these results clearly demonstrate the high quality of the RCP BB generated by our metadevice (see Fig. S15 for the intensity distributions at *z* = 3 mm and *z* = 4 mm in Supplementary Information). The agreement between the measured/simulated transverse field patterns with the theoretical curves again reinforces our claim that the generated BB is not adversely affected by interference from the normal-mode background, which can attributed to the high relative efficiency of the designed PB meta-atom.

As a comparison, we repeated the above analyses for a frequency of 0.4 THz, which is outside the working frequency band of the PB meta-atom. The results (see Fig. S17 in Supplementary Information) show that the generated BB exhibits poor quality with transverse field patterns that significantly deviate from the analytical prediction due to interference with the strong normal-mode background generated at this frequency.

As a final remark, we also retrieved the LCP field components from the experimentally measured data at the working frequency of 0.66 THz. As expected, the |*E*^+^| field distribution does not exhibit any BB features (see Fig. S16 in Supplementary Information) as the PB device is designed only for generating RCP BBs. An LCP BB generator could be easily designed by setting the rotation-angle profile as *ϕ*(*x*, *y*) = −Φ(*x*, *y*)/2.

## Discussion

To summarize, we demonstrated that high-performance manipulations of CP THz beams can be achieved by ultrathin transmissive PB metasurfaces constructed by carefully designed meta-atoms based on a freestanding ABA structure. Two effects were experimentally demonstrated by our *z*-scan measurements: a PSHE (with a relative efficiency reaching 90%) and high-quality BB generation. Other fascinating physical effects can be expected as long as appropriate metasurfaces are designed/fabricated based on such high-efficiency meta-atoms. Our results lay a solid basis to realize high-performance THz metadevices for controlling CP beams, which can be useful in versatile applications, such as biomolecular manipulations, bioimaging and THz telecommunications.

## Materials and methods

### Numerical simulations

We performed FDTD simulations numerical software Conterto 7. 0 of Vector Fields from UK. In our simulations, we used plane-wave input with periodic boundary conditions to study the Jones' matrix characteristics of the periodic sample, and plane-wave input with open boundary conditions to study the PSHE and BB generations. We treat Gold as lossy metal of conductivity 1.0*e*6 *S*/*m* in THz regime.

### Sample fabrication

Our freestanding THz tri-layer PB metasurface samples were fabricated with standard photolithography and metallization processes based on our theoretical designs. Ten-μm-thick polyimide layers were capped on the top and bottom of the metadevices to protect the sample. The periodic sample and three THz PSHE PB metasurfaces with different phase gradients (*ξ* = 0.395*k*_0_, 0.296*k*_0_, 0.222*k*_0_) have dimensions of 10 mm × 12.8 mm. The sample for CP BB generation is 3.2 mm × 3.2 mm in size with 25 by 25 meta-atoms.

### Experimental setup

We used a TDHIS, as illustrated in Fig. 3a, to perform experimental characterizations. An ultrafast 50fs laser pulse generated by a typical laser amplifier system with operating wavelength of 800 nm and repetition ratio of 1 kHz (900 mW average power) was divided into a pump beam to produce THz emission and a probe beam to measure the THz signal. Illuminated by the pump beam, the ZnTe crystal radiates THz waves via optical rectification. We use another ZnTe crystal to detect the THz signal passing through our samples. To experimentally characterize the different polarization components of THz signal, we employed a HWP and a polarizer to control the polarization of probe beam. Thanks to linear electro-optic effect in the detection ZnTe crystal, the polarization of probe beam can be modulated by the THz field to obtain two-dimensional (2D) field distribution of THz signal. Our imaging module to capture the modulated THz probe beam consists of a Wollaston prism, a quarter-wave plate, two lenses, and a CCD camera. The imaging area of CCD is 8 mm × 8 mm, corresponding to 300 × 300 pixels for each THz image. By capturing the probe beam’s image with imaging module, we can obtain the 2D THz field distribution based on balanced electro-optic detection techniques.

To characterize the PSHE performance of our THz PB metadevices, we illuminated our fabricated sample with *x*-polarized waves and measured the phase and amplitude spectrum of the Ex and Ey components of the transmissive THz wave by switching the HWP in our TDHIS. A pinhole with a diameter of 2 mm was placed before the samples to guarantee that all the THz waves had passed through it.

To experimentally demonstrate the CP BB generation, we performed a *z*-scan measurement based on our TDHIS by linearly varying our metadevice mounted on a moving stage to evaluate the longitudinal E field distributions of the generated THz BB.

## Supplementary information

Supplemental Material
