# Supplementary material for: Efficient manipulations of circularly polarized terahertz waves with transmissive metasurfaces
Source: Light Sci Appl. 2019 Jan 30;8:16. doi: 10.1038/s41377-019-0127-0 (PMC6351568; doi:10.1038/s41377-019-0127-0)
Supplement: Supplementary file 1 — Supplemental Material [file 41377_2019_127_MOESM1_ESM.docx]

**Supplementary information: Efficient manipulations of circularly polarized terahertz waves with transmissive metasurfaces**

Min Jia,^1$^ Zhuo Wang,^1$^ Heting Li^2$^, Xinke Wang,^2^ Weijie Luo,^1^ Shulin Sun,^3^ Yan Zhang,^2^ Qiong He, ^1,4*^ and Lei Zhou^1,4*^

1. State Key Laboratory of Surface Physics and Key Laboratory of Micro and Nano Photonic Structures (Ministry of Education), Fudan University, Shanghai 200438, China

2. Beijing Key Laboratory of Metamaterials and Devices, Key Laboratory of Terahertz Optoelectronics, Ministry of Education, and Beijing Advanced Innovation Center for Imaging Technology, Capital Normal University, Beijing, 100048, China

3． Shanghai Engineering Research Center of Ultra-Precision Optical Manufacturing, Green Photonics and Department of Optical Science and Engineering, Fudan University, Shanghai 200433, China

4. Collaborative Innovation Center of Advanced Microstructures, Nanjing 210093, China

$ The authors contributed equally to this work

*Corresponding authors: phzhou@fudan.edu.cn; qionghe@fudan.edu.cn

1. **Working mechanism of our high-performance meta-atom**


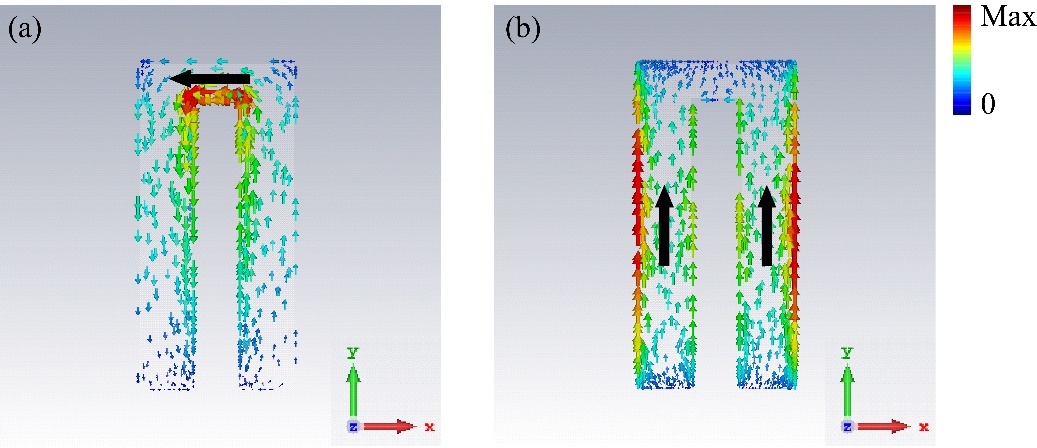


Figure S1. Simulated current distributions on a single U-shape metallic resonator, under the illuminations of (a) *x*-polarized and (b) *y*-polarized THz waves at their fundamental resonant frequencies (0.71 and 1.56 THz).

We start from illustrating the electromagnetic (EM) properties of the “modes” supported by a single U-shape resonator. Figure S1 depicts the current distributions of two *lowest-order* EM resonant modes supported by such a resonator under excitations with different polarizations. Obviously, the *x*-polarized EM wave induces currents flowing along the whole path of “U” with maximum appearing at the short bar, while the *y*-polarized excitation induces significant amount of currents mainly flowing back and forth on the two long bars. High-order modes exist for both polarizations, which were not discussed here.


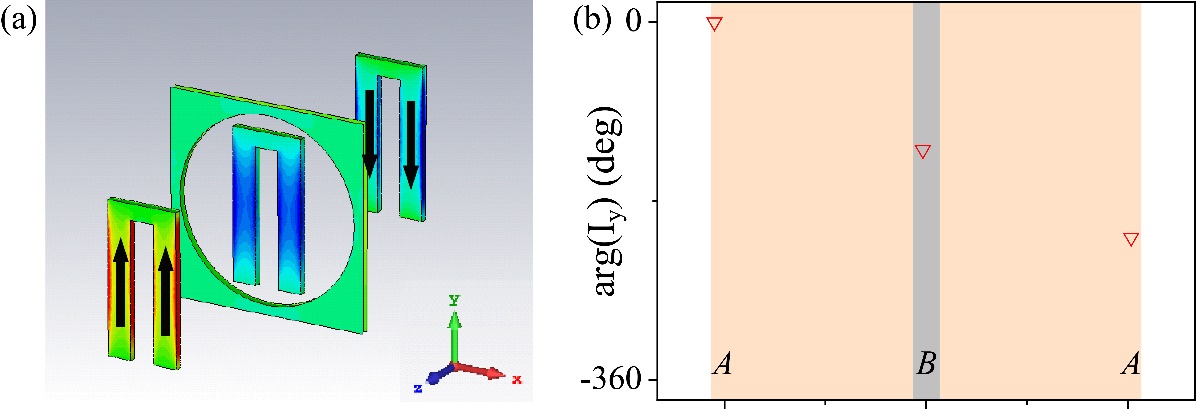


Figure S2. (a) Current distributions on different metallic layers inside the designed tri-layer structure under excitation of *y*-polarized EM wave at 0.66 THz. (b) Phases of total currents induced on three metallic layers, obtained by integrating the currents on all metallic surfaces given in FDTD simulations.

We next illustrate the physics underlying the high transmissions of our ABA structure. As we argued in the main text, generations of *effective magnetic currents* are the key to achieve the desired optical transparencies. Stacking three metallic layers together to form an ABA structure, near-field couplings between different layers can generate high magnetic fields inside the ABA structure. Taking the *y*-polarization case as an example, Fig. S2 shows that electric currents flowing on the first and third layers are nearly opposite, while that induced on the second layer is quite weak. Therefore, high magnetic fields are indeed induced inside the structure, which contribute an appropriate *effective magnetic susceptibility* $\chi_{m}$ to the whole system, finally leading to high transmissions of THz waves at particular frequencies.

**
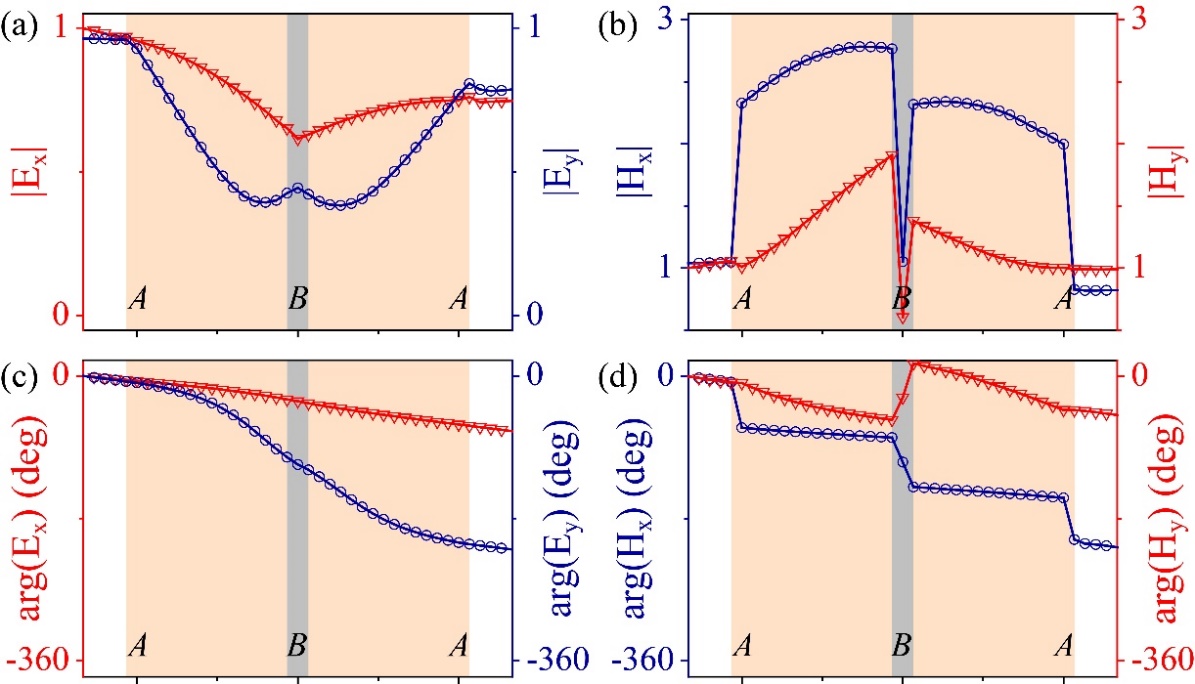
**

Figure S3. Evolutions of the electric (a, c) and magnetic (b, d) fields inside the designed tri-layer structure illuminated by normally incident EM waves polarized along *x* and *y* axes calculated by FDTD simulations at 0.66 THz. Field integrations over the *x-y* plane within a unit cell are performed to obtain the results.

Such an argument is reinforced by Fig. S3, where we illustrated how the amplitude and phase of electric and magnetic fields (*averaged* over *x-y* planes at different *z*) evolve inside such an ABA structure, which is shined by normally incident EM waves with two polarizations. Indeed, EM waves with both polarizations can transmit efficiently through the meta-atom, but the accumulated phases of the transmitted waves exhibit a difference of π. The transmission coefficients do not reach 100% because losses exist inside the structure. The most important message conveyed by Fig. 3S is that significant magnetic fields are induced inside the structure, which are primarily responsible for the enhanced high transmissions as we argued in the main text.


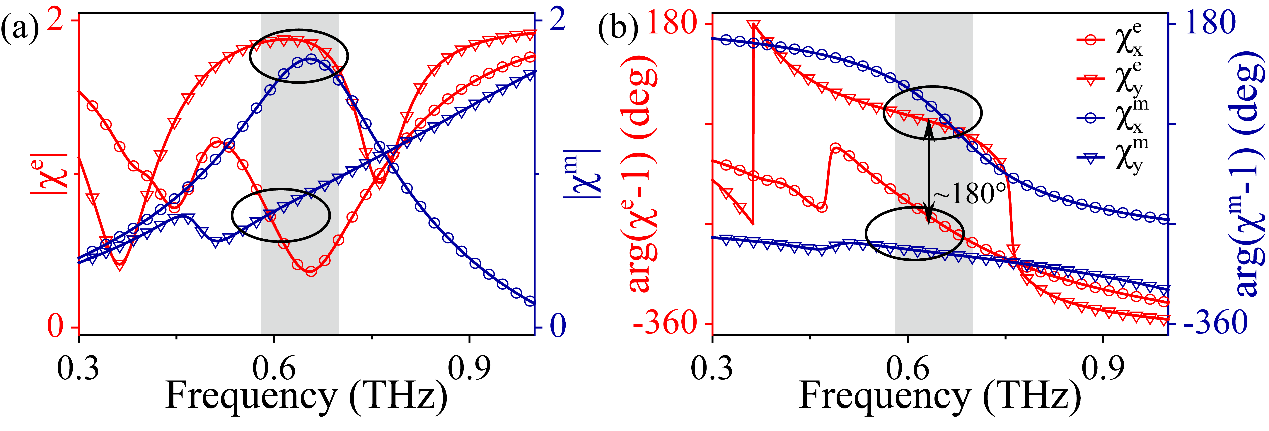


Figure S4. Spectra of (a) amplitudes and (b) phases of the electric and magnetic susceptibilities of our meta-atom obtained by integrating FDTD-simulated electric or magnetic current distributions inside the structure illuminated by normally incident plane waves with polarizations along the x and y axes.

To quantify such a physical argument, we follow Ref [31] to quantitatively evaluate the effective electric and magnetic susceptibilities of the whole structure, through integrating electric and magnetic currents induced in the structure obtained by FDTD simulations at different frequencies. Figure S4 shows how these effective susceptibilities (i.e., $\chi_{x}^{e},\chi_{y}^{e},\chi_{x}^{m},\chi_{y}^{m}$) vary against frequency. Clearly, inside the working band, the system exhibits nearly air-matched impedance for two polarizations (see Fig. 4(a)), but with phases roughly satisfying the desired condition $\chi_{x}^{e}=\chi_{y}^{m}=1+e^{i\delta},\text{ }\chi_{y}^{e}=\chi_{x}^{m}=1+e^{i(\delta+\pi)}$ as given by Ref. [31]. Note that due to material losses (both from dielectrics and metals) inevitably existing in this frequency domain, we can only make the 100%-efficiency condition (derived in lossless case) *approximately* satisfied (see Fig. 4). This is also the inherent reason why we need a more complexed U-shape resonator to design our ABA meta-atom, since we naturally need more freedoms in optimizations.


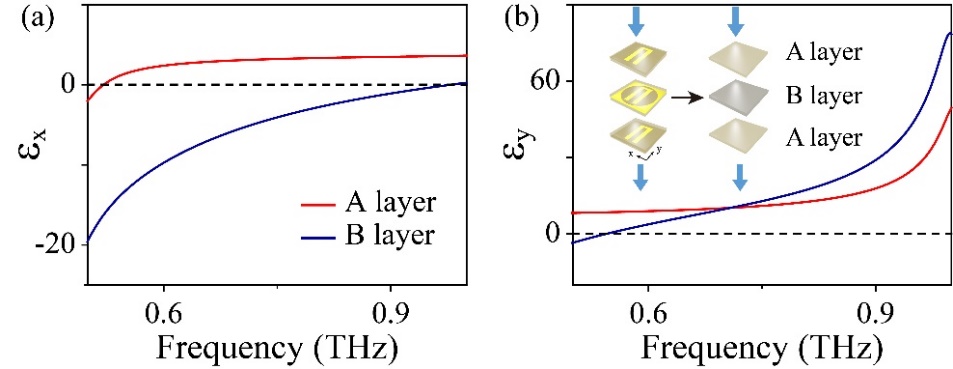


Figure S5. Effective permittivity of two layers for two polarizations as functions of frequency, retrieved from FDTD simulated transmission spectra, based on the standard S-parameter retrieval method. Here the thicknesses of A-layer and B-layer are 25.065 μm and 30.065 μm, respectively.

Finally, we discuss more detailed physical mechanisms underlying the high transmissions for two polarizations. If we treat the ABA structure as a whole, then the physics accounting for them are all due to enhanced magnetic responses (see Fig. S3-4). However, subtle differences exist if we *do not* treat the ABA structure as a whole, but rather only homogenize each layer as an effective-medium slab (see inset to Fig. S5(b)). In that case, our previous analyses [31] already showed that, such ABA structure (with effective permittivity of two layers given by $\varepsilon_{A}$ and $\varepsilon_{B}$, respectively) naturally supports high transmissions based on two different mechanisms (see Fig. 2 in Ref. [31]). When both $\varepsilon_{A}$ and $\varepsilon_{B}$are positive, the transparency is governed by Fabry-Perot (FP) resonance, while the mechanism turns to be scattering cancellations when $\varepsilon_{A}$ and $\varepsilon_{B}$ exhibit different signs. To clarify the mechanisms in our case, we retrieved the effective permittivity of two layers from FDTD simulated transmission spectra, based on the standard S-parameter retrieval method. Figure S5 depicts the computed effective permittivity of two layers for two polarizations. Within the working frequency band, whereas the transparency for *x*-polarization is governed by scattering cancellation mechanism since the permittivity of two layers are of different signs, the transparency for *y*-polarization must be due to FP resonance since both $\varepsilon_{A}$ and $\varepsilon_{B}$ are positive, It is such difference in mechanisms that leads to the significant difference in transmission phases for two polarizations, which eventually help us achieve the desired half-wave-plate design.

1. **Simulated transmission properties of an optimized meta-atom based on single bar-resonator**


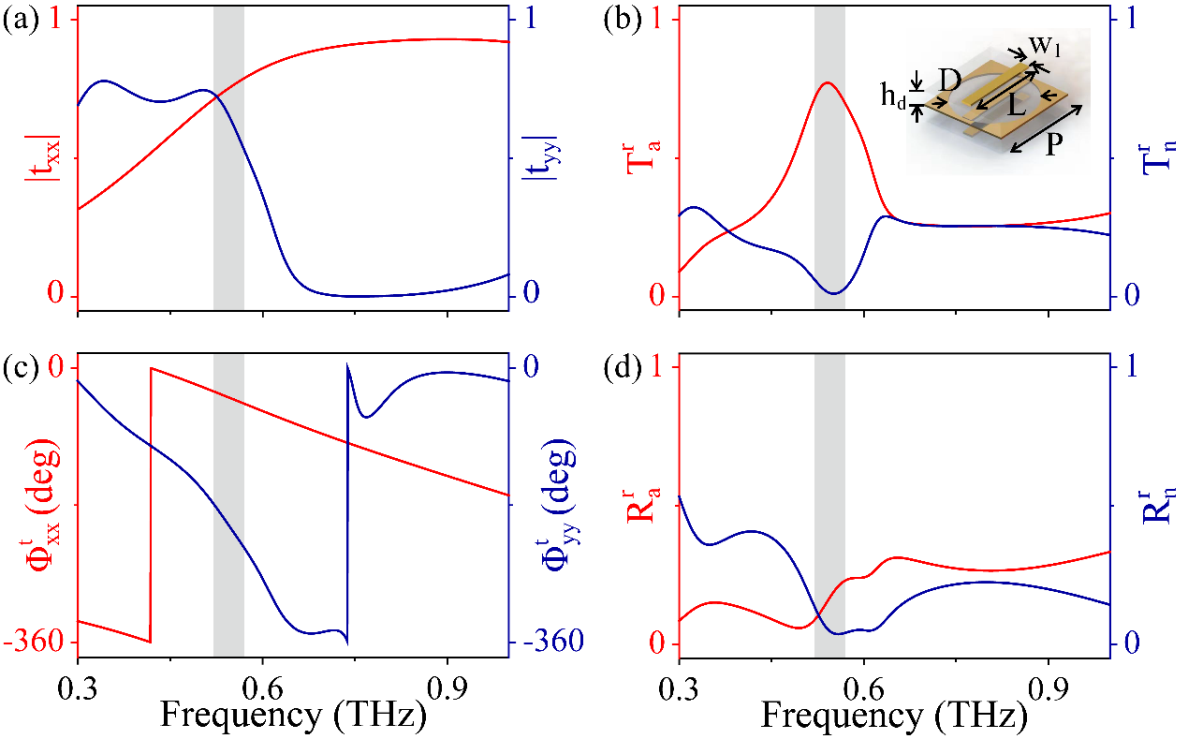


Figure S6. FDTD simulated spectra of transmission (a) amplitude and (c) phase for a meta-atom based on bar-resonator under excitations of x- polarized (red) and y-polarized (blue) THz waves. Spectra of (b) $T_{a}^{r},T_{n}^{r}$ and (d) $R_{a}^{r},R_{n}^{r}$ of the PB-atom retrieved from simulated results on Jones’ matrix characteristics. Inset: Schematics of the tri-layer meta-atom based on bar-resonator with following geometrical parameters: $D=116um$,$h_{\text{d}}=30um$ $p=128um,w=15um,L=112um.$.

To identify the advantages of using a complex “U”-shape resonator in designing our THz meta-atom as compared to previous microwave design based on a bar resonator, we performed FDTD simulations to study the EM properties of a (optimized) meta-atom based on single-bar resonators. FDTD simulations show that such tri-layer meta-atom can also achieve the desired half-wave-plate functionality with reasonably high transmissions for both *x*- and *y*- polarized beams. However, compared to the one realized with the “U”-shape structure (see Fig. 2 in the main text), it exhibits relatively narrower working bandwidth (relative bandwidth 9%), obviously due to lacking of geometrical freedoms in the optimization processes. In fact, a “U”-shape resonator supports more EM modes than a bar-resonator, which can help us in optimizing the performance of the THz design.

1. **Measured and simulated reflection properties of the designed meta-atom**

**
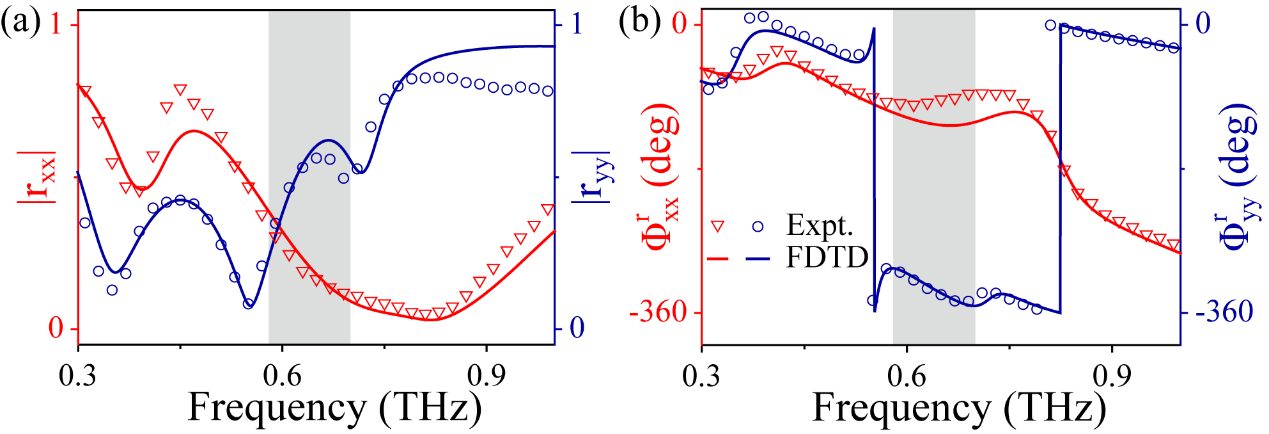
**

Figure S7. Measured and simulated spectra of reflection amplitude (a) and phase (b) for the fabricated periodic sample as studied in Fig. 2.

1. **Influences of material losses on the properties of the designed meta-atom**


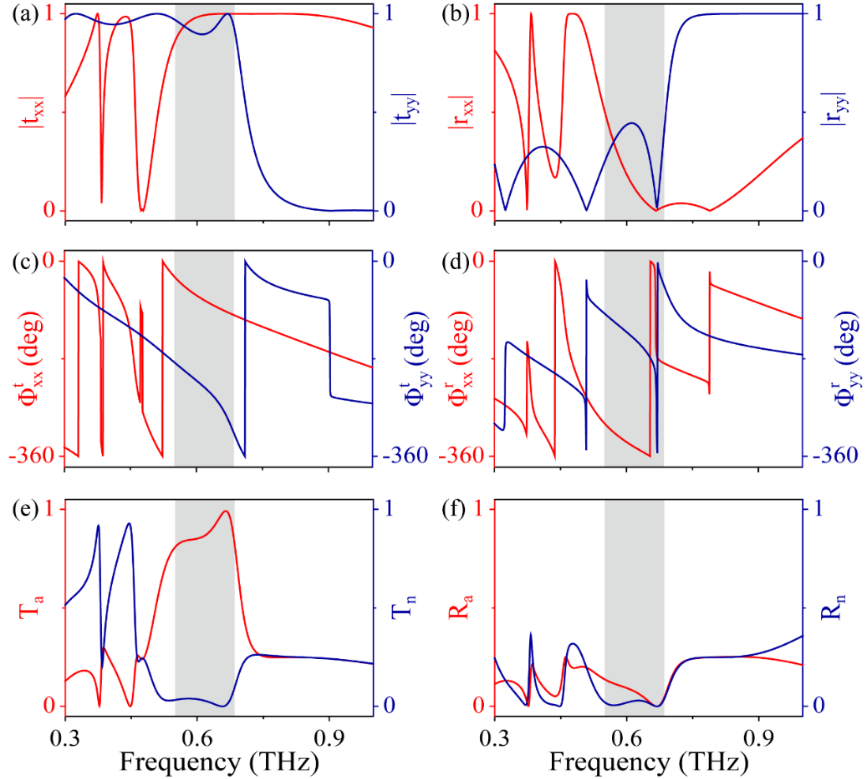


Figure S8. FDTD simulated spectra of transmission/reflection amplitude (a/b) and phase (c/d) for an optimized meta-atom in the *completely lossless* case (assuming that losses in both metals and dielectrics are zero). Efficiency spectra of $T_{a},T_{n}$ (e) and $R_{a},R_{n}$ (f) for the PB metasurface with the ideal meta-atom calculated with Eq. (2) based on simulated transmission and reflection properties of the meta-atom presented in (a-d). Geometry parameters of the optimized lossless meta-atom: $D=116um,$ $p=118um,$ $L=100um,$ $w_{1}=12um,w_{2}=42um,$ $h_{d}=45um,h_{m}=65nm$.

We first study the ideal case where losses are dropped in both metals and dielectrics. In this case, our calculations (Fig. S8) show that we can indeed design a meta-atom exhibiting 100% efficiency.

We next compare the performances of two meta-atoms (Fig. S9), in which metals are always assumed as realistic lossy metals but dielectrics are assumed to either have losses or not. We find from Fig. S9 that dielectric losses can hardly change the performance of the meta-atom, as long as the metals are lossy.

**
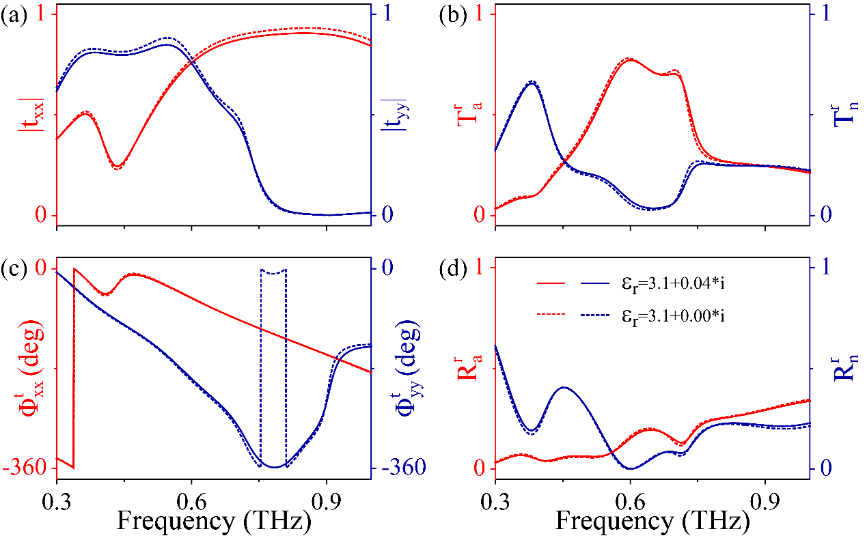
**

Figure S9. FDTD simulated spectra of transmission (a) amplitude and (c) phase for the designed tri-layer meta-atom corresponding to the dielectric layer with (solid line) and without (dash line) absorption. Spectra of (b) $T_{a}^{r},T_{n}^{r}$ and (d) $R_{a}^{r},R_{n}^{r}$ of the PB-atom retrieved from simulated results on Jones’ matrix characteristics.

**
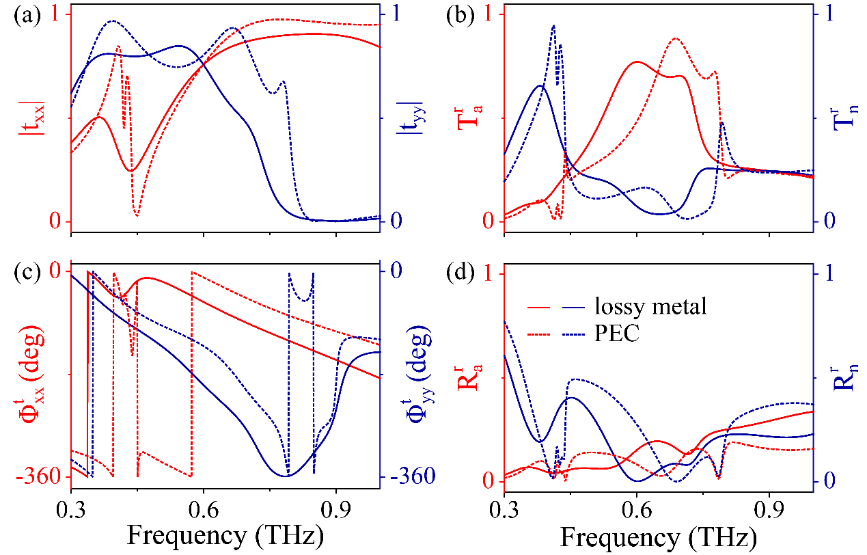
**

Figure S10. FDTD simulated spectra of transmission (a) amplitude and (c) phase for the designed tri-layer meta-atom corresponding to the gold treated as lossy metal (solid line) and PEC (dash line). For these two cases, the dielectric layer is lossy ($\varepsilon_{r}=3.1+0.04*i$). Spectra of (b) $T_{a}^{r},T_{n}^{r}$ and (d) $R_{a}^{r},R_{n}^{r}$ of the PB-atom retrieved from simulated results on Jones’ matrix characteristics.

We finally compare the performances of two meta-atoms (Fig. S10), in which dielectrics are always assumed as lossy material but metals are assumed as either lossy metals or perfect electric conductors (PEC). We find in such case (Fig. S10) losses in metals can significantly change the performance of the meta-atom. From these comparisons, we can draw a conclusion that losses in metals play more important roles to degrade the performance of our meta-atom.

1. **Originally measured E-field distributions in LP bases for the** $\xi=0.296k_{0}$**PB metasurface at 0.66 THz**

**
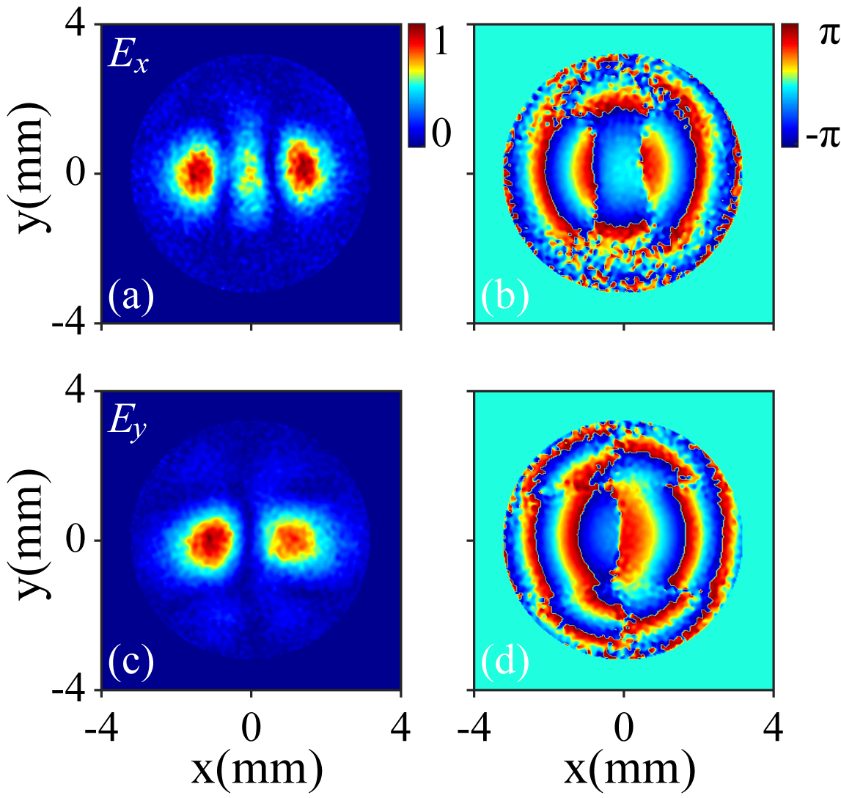
**

Figure S11: Measured amplitude (a,c) and phase (b,d) distributions of Ex (up-panel) and Ey (bottom panel) components on the *x-y* plane with z = 3.5mm for the $\xi=0.296k_{0}$PB metasurface (as studied in Fig. 3) shined by *x*-polarized THz wave at 0.66THz.

1. **Sizes of the input THz beams at different frequencies**

The incident THz beam exhibits a different size at different frequencies due to the presence of a pinhole placed in front of the sample. Figure S12 illustrates the measured amplitude and phase distributions of $E_{x}$ component on the target x-y plane for incident THz waves (with the sample taken away) at different frequencies. One can notice that the beam size of the THz beam is a decreasing function of frequency, due to the diffractions at the pinhole.


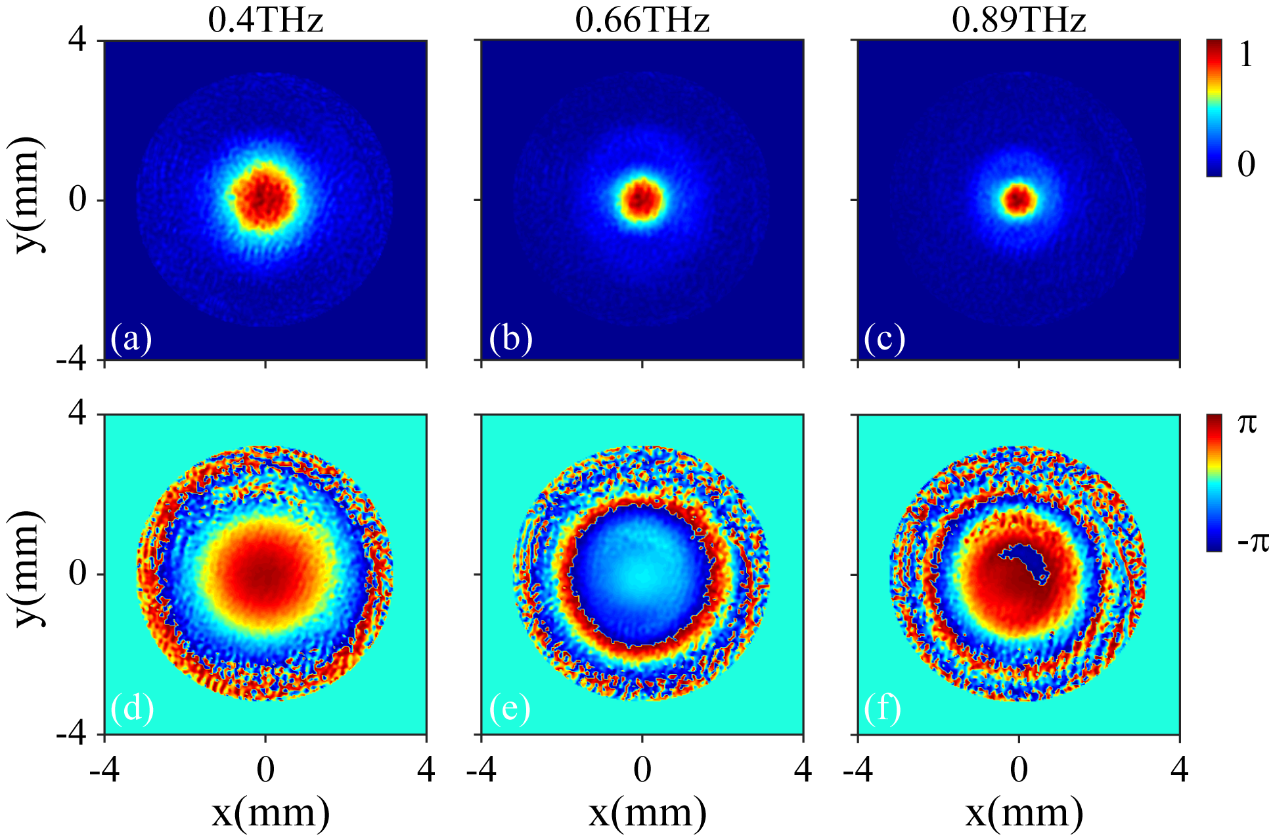


Figure S12. Measured amplitude (a-c) and phase (b-d) distributions of $E_{x}$ component at the target x-y plane, for input THz beams at three different frequencies.

1. **Simulation and experimental results of PSHE at PB metasurfaces with different phase gradients**


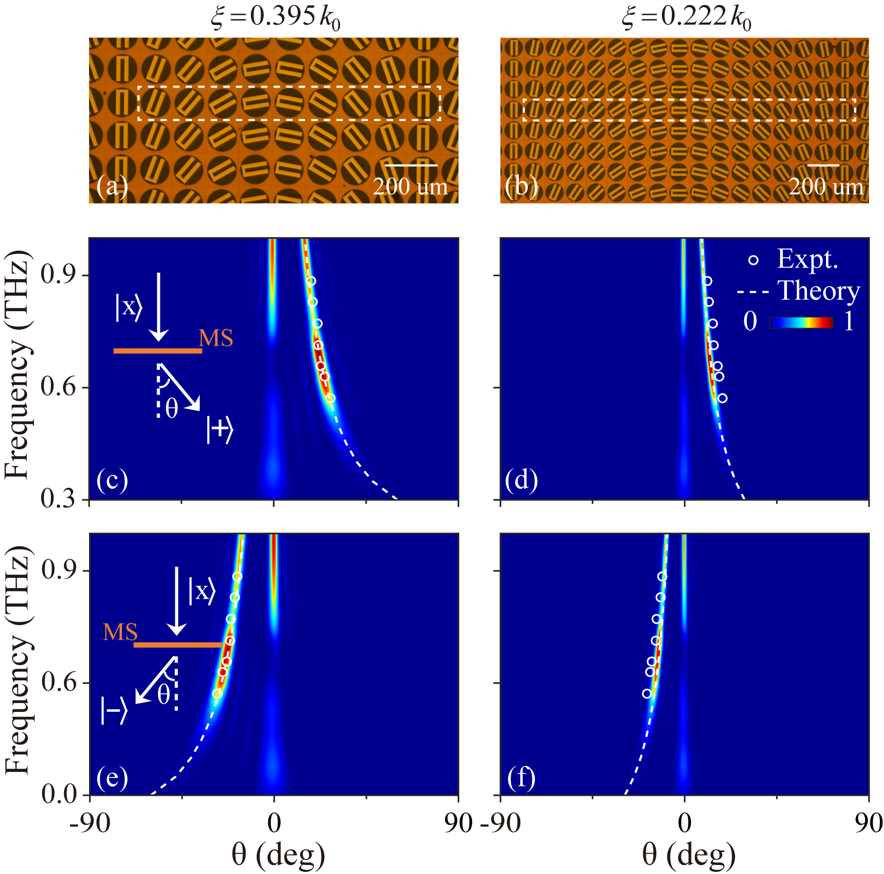


Figure S13. Macrographs of fabricated PB metasurfaces with $\xi=0.395k_{0}$ (a) and $\xi=0.222k_{0}$(b), respectively. FDTD simulated scattered-field intensity (color map) of the transmitted LCP wave (c, d) and RCP wave (e, f) versus frequency and deflection angle for the corresponding meta-devices, respectively, under illumination of normally incident x-polarized THz beams. White circles and dashed line in (c-f) represent the experimental results and theoretically calculated results based on generalized Snell’s law with Equation 3.

**
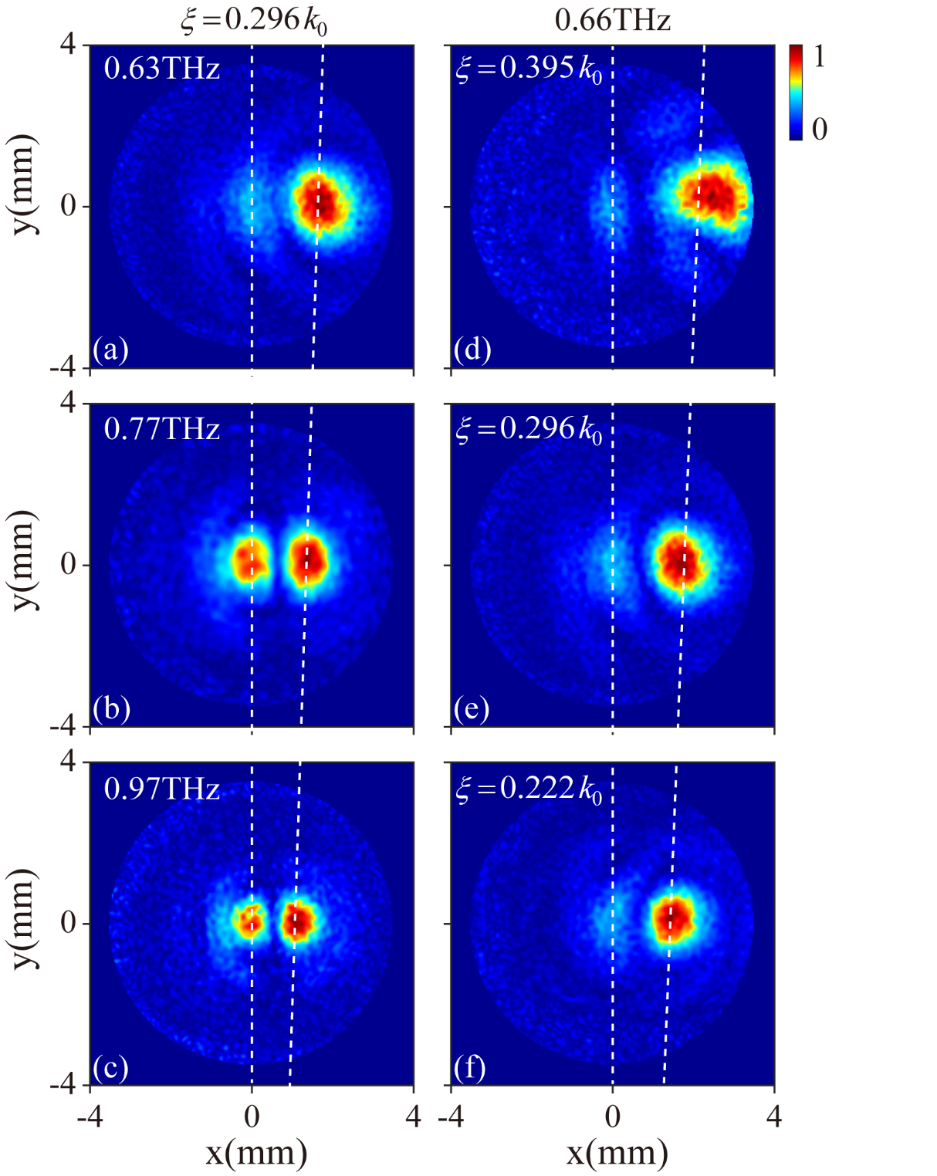
**

Figure S14. (a-c) Measured field distribution of transmitted LCP THz beam through the fabricated PB metasurface with $\xi=0.296k_{0}$ at 0.63 THz, 0.77 THz and 0.97 THz, respectively. (d-f) Measured field distribution of transmitted LCP THz beam at working frequency of 0.66THz through the three fabricated PB metasurfaces with $\xi=0.395k_{0}\text{， 0.296}k_{0}\text{， }0.222k_{0}$, respectively.

Figures S14 (a-c) clearly shows that the deflection angle is a decreasing function of frequency for a PB metasurface with a given phase gradient, in consistency with the prediction of the generalized Snell’s law (Eq. (3) in the main text). Meanwhile, at a given frequency, the anomalous deflection angle is an increasing function of the phase gradient $\xi$ as shown in Fig. S14 (d-f), which is again consistent with the generalized Snell’s law.

1. **Verifications on the fidelity of the generated BBs**


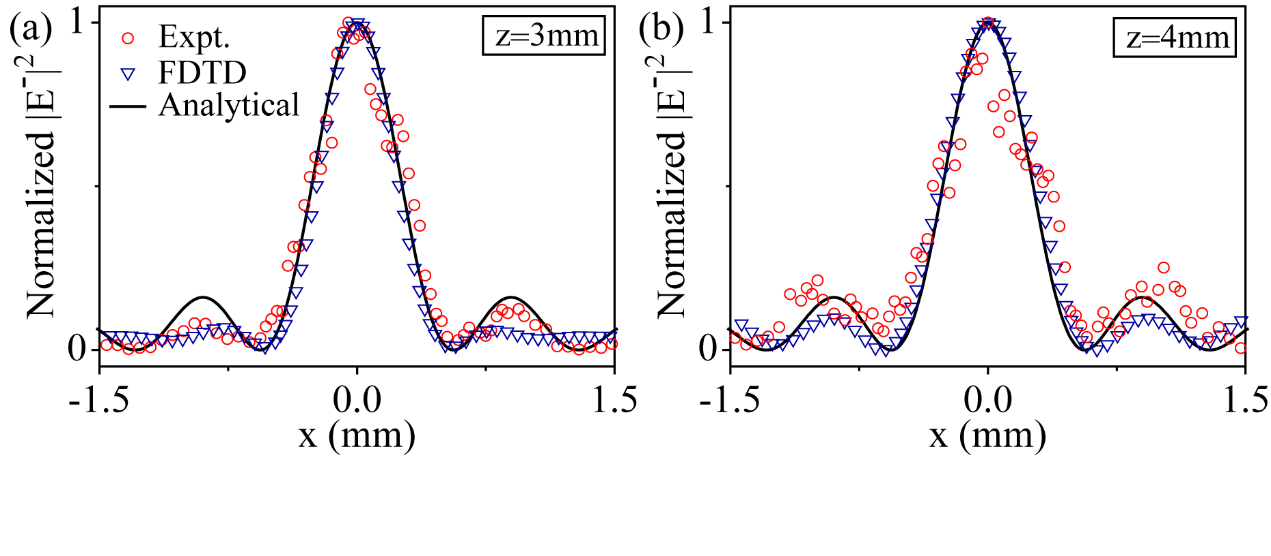


Figure S15. Normalized $\left|E^{-}\right|^{2}\sim x$ distributions at 0.66 THz along the line with z=3mm, y=0mm (a) and z=4mm, y=0mm (b), respectively, obtained by experiment (red circles), FDTD simulations (blue triangles) and theoretical formula for zero-order BB (solid line). All the data are normalized against its own maximum.

1. **Simulated and measured E-field distributions of the transmitted LCP beam passing through our BB generator at 0.66 THz**


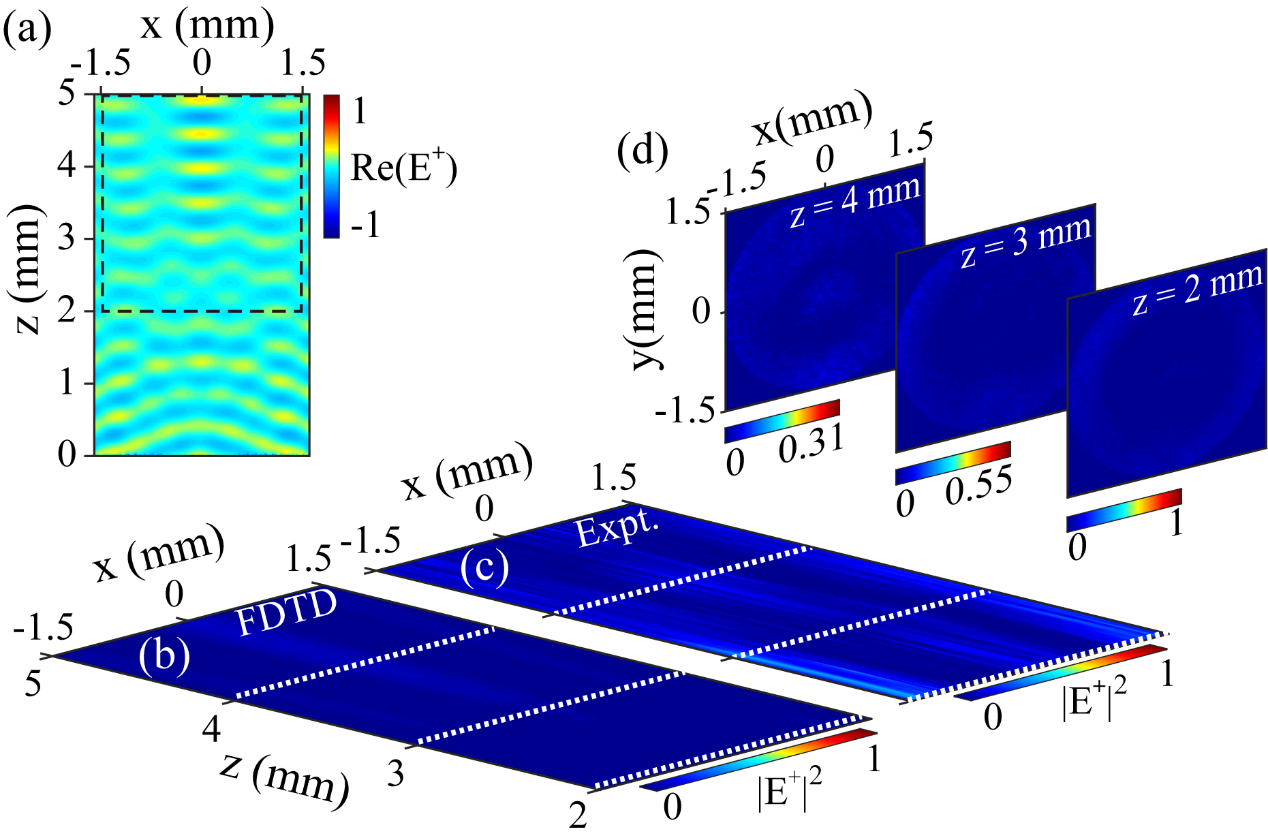


Figure S16. (a) FDTD simulated Re($E^{\text{+}}$)distribution of our meta-devices in x-z plane under normal-incidence of x-polarized THz wave at 0.66THz. FDTD simulated (b) and Z-scan measured (c) $\left|E^{+}\right|^{2}$ distribution (color map) of the transmitted wave in x-z plane under illumination of normally incident x-polarized THz wave at 0.66 THz, respectively. (d) Measured$\left|E^{+}\right|^{2}$ distribution (color map) of the transmitted wave at 0.66 THz in x-y plane at the position of z=2 mm, 3 mm and 4 mm. All the data are normalized against its maximum value obtained for $\left|E^{-}\right|^{2}$ distribution shown in the main text.

1. **Simulated and measured performance of the fabricated BB generator at 0.4 THz**


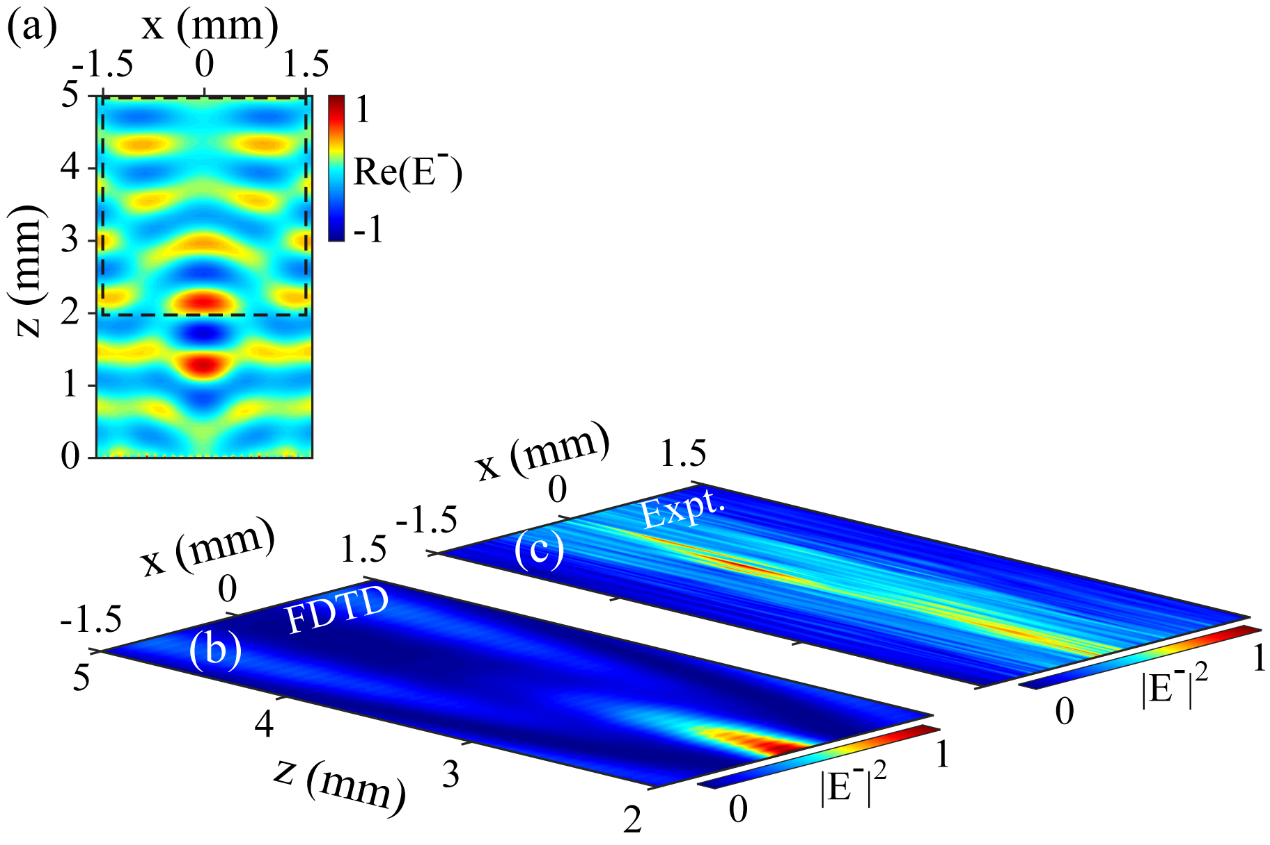


Figure S17. (a) FDTD simulated Re($E^{-}$)distribution of our meta-devices in x-z plane under normal-incidence of x-polarized THz wave at 0.4THz. FDTD simulated (b) and z-scan measured (c) $\left|E^{-}\right|^{2}$ distribution (color map) of the transmitted wave in x-z plane under illumination of normally incident x-polarized THz wave at 0.4 THz, respectively.
